# Melanoma resists chemotherapy through an adaptive mitochondrial response

**DOI:** 10.1186/s13046-026-03685-8

**Published:** 2026-03-26

**Authors:** Mehrdad Zarei, Semmer A. Ali, Alexander W. Loftus, Faith Nakazzi, Hallie J. Graor, Om Prajapati, Sami O. Abul-Khoudoud, John M. Asara, Rui Wang, Jordan M. Winter, Luke D. Rothermel

**Affiliations:** 1 Case Comprehensive Cancer Center, Case Western Reserve University, Cleveland, OH USA; 2 Department of Surgery, Division of Surgical Oncology, University Hospitals Cleveland Medical Center, OH Cleveland, USA; 3 Department of Medicine, Harvard Medical School, Boston, MA USA

**Keywords:** Melanoma, Metabolic reprogramming, Mitochondria, TCA cycle, OXPHOS, ETC, Complex I, Chemosensitivity, Combination therapy

## Abstract

**Background:**

Malignant melanoma is one of the most common types of cancer in the United States. Despite improvements in therapeutics, advanced disease is often lethal if the cancer develops resistance to immune and targeted therapies. Metabolic reprogramming is an important hallmark of cancer and drives resistance to certain therapies. Previous efforts to modulate melanoma metabolism have focused on targeting glycolysis, whereas targeting mitochondrial oxidative phosphorylation (OXPHOS) and the TCA cycle is relatively less developed for melanoma.

**Methods:**

Melanoma cell lines were evaluated under chemotherapy-associated stress using assays of mitochondrial function and oxidative metabolism. Pharmacologic inhibition of the mitochondrial electron transport chain was assessed using complex I inhibitors in combination with conventional anti-melanoma chemotherapy in preclinical in vitro models, with validation in animal models.

**Results:**

Melanoma cells rely heavily on mitochondrial function, particularly under chemotherapy-associated stress, as evidenced by increased OXPHOS activity. Pharmacologic inhibition of the mitochondrial electron transport chain (ETC) with complex I inhibitors (Phenformin and IACS-010759) synergizes with conventional anti-melanoma chemotherapy in pre-clinical in vitro models of melanoma, with added benefit in animal models.

**Conclusions:**

These findings suggest that melanoma adapts to chemotherapy by increasing mitochondrial oxidative metabolism. Targeting the ETC offers a compelling strategy to enhance chemotherapy activity in patients with advanced and treatment-refractory melanoma.

**Graphical Abstract:**

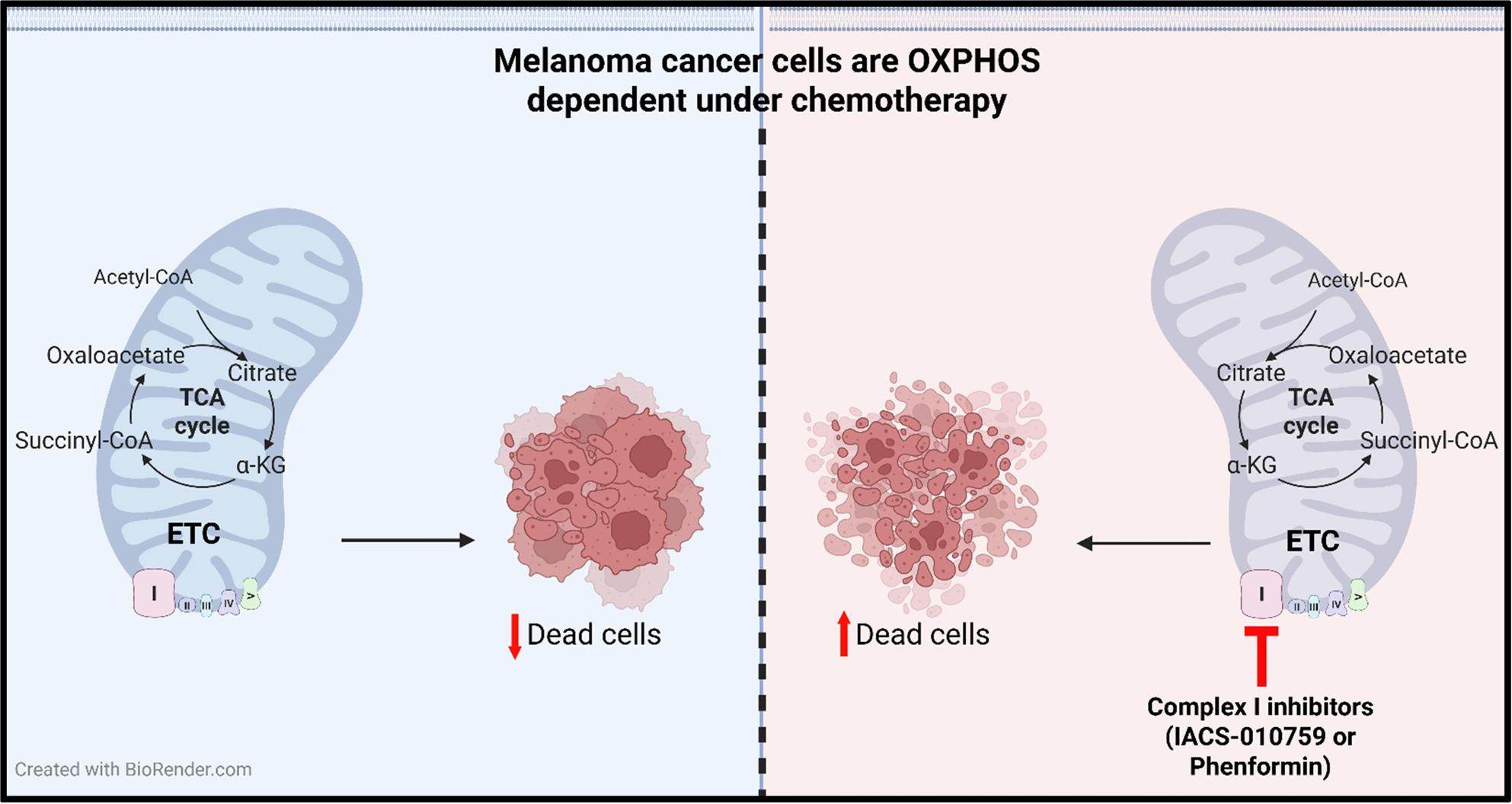

**Supplementary Information:**

The online version contains supplementary material available at 10.1186/s13046-026-03685-8.

## Background

Modern checkpoint inhibitors (ICIs) immunotherapy and BRAF/MEK targeted therapies have revolutionized the treatment of advanced melanoma. The current first-line therapy for most patients with metastatic melanoma consists of checkpoint inhibitors, nivolumab (anti-PD1 antibody) and ipilimumab (anti-CTLA4 antibody). Median overall survival with this approach is estimated to be 71.9 months, and 10-year survival rates approach 37% [1–5].

Despite these remarkably improved outcomes over the past 15 years, 40% of patients do not respond to immune therapies and 60% experience significant toxicities. Responses are also not durable for many patients; tumors in one-third of responders (20% overall) eventually acquire resistance [3, 6, 7]. Targeted therapies such as BRAF and MEK inhibitors have shown success in BRAF mutated tumors in the first- and second-line settings, with a positive impact on progression-free and overall survival [8]. However, only 40–50% of melanoma tumors carry BRAF mutations [9, 10], and resistance to treatment reliably occurs, with a median progression-free survival of only 11–12 months [9, 11]. Due to the progress seen with immunotherapy and targeted therapy drugs over the past decade, alternative biologic pathways that can be successfully targeted have received comparatively less attention from the melanoma research community [12].

Prior to the introduction of modern therapeutics, systemic chemotherapy was the first line option for metastatic melanoma. Dacarbazine is the only FDA-approved chemotherapeutic for melanoma. Treatment of advanced disease offers a marginal 13% response rate and 5-year overall survival of 6–9% when used as monotherapy [13, 14]. No significant increase in overall survival was described with multi-agent chemotherapy regimens, including additional agents like bleomycin, vincristine, lomustine, and dacarbazine with interleukin-2 [15]. Dacarbazine is a prodrug of the alkylating agent 5-(3-methyltriazen-1-yl) imidazole-4-carboxamide (MTIC) that methylates guanine residues and triggers cell apoptosis [15]. Temozolomide (TMZ) is similarly a prodrug of MTIC, but has the advantages of better blood-brain barrier penetration and oral delivery making this a preferred choice in the limited scenarios when chemotherapy is utilized for advanced melanoma in the modern era [14, 16]. Similarly, other chemotherapies like the platinum-based, cisplatin, have been explored in melanoma treatment, but also have been limited by significant drug resistance and poor efficacy [17]. These data contribute to the perception that melanoma is generally “chemoresistant”, with implicated mechanisms for resistance including dysregulated DNA repair pathways and adaptations to oxidative stress [18–20]. While chemotherapy has been relegated to the second or third line and is rarely studied today as a bona fide melanoma treatment, in fact it is estimated that 10–20% (total of about 7,500 − 15,000) of all patients with advanced melanoma will receive chemotherapy at some point in their disease course [21, 22]. Thus, in the absence of novel therapies or improvements in existing therapies, there remains substantial opportunity to improve outcomes by improving chemotherapeutic efficacy.

Our prior work demonstrated that melanoma metabolism likely plays a role in the adaptive survival of these tumors to chemotherapy [23]. Like many tumors, melanomas outgrow their blood supply, leading to nutrient limitation, hypoxia, and tumor necrosis [24–27]. One metabolic adaptation to overcome these conditions is an increased dependence on mitochondrial oxidative phosphorylation (OXPHOS) for efficient energy production to power survival pathways when nutrients are scarce [28]. Mitochondria are multi-membrane respiratory structures that generate reducing equivalents in the tricarboxylic acid (TCA) cycle that pass through the electron transport chain (ETC) to produce ATP [29]. When mitochondria are dysfunctional, they fail to keep up with the energy demands required for cell survival under stress, and also generate toxic amounts of reactive oxygen species (ROS) due to poor handling of electrons through the ETC [30, 31]. Thus, robust mitochondria are a metabolic requirement for melanoma survival in the face of oxidative insults like chemotherapy.

Melanoma’s apparent dependency on mitochondrial OXPHOS makes various components of respiration promising targets for therapy in melanoma. Mechanisms for the direct anti-tumor effects of metformin include inhibition of mTOR, primarily through activation of AMPK. Additionally, the drug blocks c-MYC protein expression [32–34]. Phenformin, another biguanide, has been shown to have even greater affinity for complex I and synergizes with BRAF inhibitors in melanoma [35, 36]. IACS-010759, a more recently developed selective complex I inhibitor, has shown promise in preclinical studies as an effective agent against cancer, including melanoma, by disrupting mitochondrial function and inducing oxidative stress, similar to other mitochondrial-targeting agents [37]. We hypothesize that these mitochondrial inhibitors are active against melanoma and can potentiate the efficacy of chemotherapy due to the role of mitochondria in melanoma resistance to these drugs.

## Materials and methods

### Cell lines and cell culture

Human melanoma cell lines, A375, SK-MEL-28, MeWo, Hs 852.T, SK-MEL-2, and 1205Lu, were obtained from the American Type Culture Collection (ATCC), WM164 and 451Lu were sourced from Rockland, Inc. The murine melanoma cell lines YUMM1.7 and B16-F10 were also acquired from ATCC. Cells were cultured in high glucose DMEM without pyruvate (Life Technologies, 11965-118), supplemented with 1% penicillin-streptomycin (Invitrogen), and 10% FBS (Gibco/Invitrogen), at 37 °C in 5% humidified CO_2_ incubators. Cell lines were treated with prophylactic doses of Plasmocin (Invivogen, #MPP-46-04) and tested for Mycoplasma (#MP0035, Sigma Aldrich; #LT07-318, Lonza) and were passaged at least twice before experimental use.

### Cell viability assays

Cells were seeded in 96-well plates at 1,500–3000 cells per well, and after 24 h, and were treated with TMZ (Sigma-Aldrich, T2577) and ETC inhibitors including phenformin hydrochloride (MedChemExpress, HY-16397 A), at the mentioned concentrations. All experiments for cell viability lasted for 5 days unless otherwise detailed. Cell viability was quantified using the Quant-iT^™^ PicoGreen^™^ dsDNA assay kits (Invitrogen; P7589).

Drug combination assays were performed by seeding 1,500–3000 cells per well in 96-well plates. After 24 h, cells were treated with different concentrations of TMZ (dose range, 50–800 µM/mL), phenformin (dose range, 0.3–40 µM/mL), in a 6 × 8 dosing matrix. Each treatment was done in triplicate. Cell growth relative to vehicle treatments was measured with Quant-iT PicoGreen [38, 39].

Drug interaction data were quantified and classified as synergistic, additive, or antagonistic using the Bliss Independence model, as previously described [40]. The combined effect of drugs X and Y was calculated using the following equation: $Effect\;X+Y\left[\left(Effect\;X+Effect\;Y\right)-\left(Effect\;X\times\;Effect\;Y\right)\right]$, where 𝑋 and 𝑌represent two different drugs. The synergy scores were interpreted as follows:

Synergy: The observed effect is greater than expected (> 0).

Additivity: The Observed effect is about the same as expected (≈ 0).

Antagonism: The observed effect is less than expected (< 0).

### Real-time quantitative PCR

Total RNA was extracted using the RNeasy PureLink RNA isolation (Life Technologies; 12183025) according to manufacturers’ instructions. RNA was converted to cDNA using a High-Capacity cDNA Reverse Transcription Kit following the manufacturer’s protocol (Applied Biosystems; 4387406). qPCR was performed using TaqMan Universal Master Mix (Thermo Fisher Scientific; 4440038), along with probes for NDUFB8 (Hs07286770_g1) NDUFS6, (Hs00190035_m1) and 18 S (Hs99999901_s1) (Thermo Fisher Scientific). Analyses were performed using the Bio-Rad CFX96 Maestro Manager 2.0 software.

### Immunoblotting

Tumor samples were homogenized in 1X RIPA buffer containing protease inhibitors (Thermo Fisher, WC320075) on ice. Cell pellets were lysed under the same conditions. The BCA Protein Assay kit (Thermo Fisher Scientific, 23225) was used to quantify the protein concentration. Equal amounts of total protein were added to a mixture of reducing agent and loading dye (Invitrogen) and incubated at 85 °C for 10 min. Lysates were separated by size, on a 4–12% Bis-Tris gel (Thermo Fisher, NM04120), using electrophoresis, and transferred to a PVDF membrane. The membranes were blocked in 5% skimmed milk dissolved in TBS-T and probed overnight at 4 °C with primary antibodies. Probed proteins included TOM20 (Cell Signaling, 13929) and β**-**Actin (Santa Cruz, sc-47778), Cleaved Caspase-3 (Cell Signaling, 9661) and γ-H2AX (Cell Signaling, 9718T). Western blotting membranes were treated with Enhanced Chemiluminescence reagent (Super Signal West Pico Chemiluminescent substrate, Thermo Fisher Scientific) and bands were detected by autoradiography (Thermo Fisher Scientific).

### Metabolite quantitation by LC-MS/MS

To harvest intracellular metabolites, cells were seeded 1 × 10^4^ complete growth medium in 10 cm plates, in biological sextuplicates. After cells were grown to ~ 60% confluence, they were treated with the indicated drug in media for 48 h. After 48 h, the media was removed and rinsed with ice-cold PBS. Metabolites were extracted using ice-cold 80% high-performance liquid chromatography (HPLC)-grade methanol on dry ice by scraping cells from each plate. The cells were dried by speed vac and frozen at -80 °C. Samples were resuspended in HPLC-grade water and liquid chromatography-mass spectrometry was performed.

Polar metabolites were quantified by 5500 QTRAP triple quadrupole mass spectrometry (AB/SCIEX) coupled to a Prominence UFLC HPLC system (Shimadzu) using amide HILIC chromatography (Waters) at pH 9.2, as previously described [41]. 299 endogenous water-soluble metabolites were measured at a steady state.

### Mitochondrial respiration

Melanoma cells (A375, SK-MEL-28 and 1205Lu) were seeded at 1 × 10^4^ cells/well in Agilent XFp Cell Culture 8-well Poly-D-Lysine pre-coated plates (#103025-100) in complete media (25 mM Glucose and 2 mM Glutamine) at 37 °C in 5% humidified CO_2_ incubators. Oxygen consumption rates (OCR) were performed using the XFp mini extracellular analyzer (Seahorse Bioscience). Cells were treated with TMZ or vehicle for 36 h. One day prior to experiments, XFp FluxPak cartridges (#103022-100), were hydrated using the XF Calibrant (#100840-000) and incubated overnight at 37 °C using a non-CO_2_ incubator. The next day cells were washed twice and media were replaced by Seahorse XF base phenol red-free media and the plate was incubated at 37 °C in a humidified atmosphere and in a non-CO_2_ incubator for 45 min. The OCR was analyzed under basal condition and in response to sequential pneumatic injections of mitochondrial inhibitors, including: oligomycin (1.5 µM), FCCP (carbonyl cyanide p-trifluoromethoxyphenylhydrazone) (2.0 µM), and rotenone/antimycin A (each 0.5 µM) using the Seahorse Mito Stress test kit (#103015-100). Raw data were normalized to total cell numbers, as determined by Quant-iT PicoGreen^™^ (Invitrogen). Melanoma cell lines were classified as glycolytic-driven or OXPHOS-driven based on basal OCR and ECAR. For each cell line, the median OCR and ECAR values across three replicates were used to calculate the OCR/ECAR ratio. The median OCR and ECAR values across all tested cell lines were used as thresholds to define “high” versus “low” activity for each parameter. Cell lines with an OCR/ECAR ratio greater than 1 were designated as OXPHOS-driven, whereas those with a ratio less than 1 were designated as glycolytic-driven. Cell lines with a ratio approximately equal to 1 were considered dual-driven.

### Mitochondrial membrane potential and mitochondrial mass

The mitochondrial membrane potential (ΔΨm) was analyzed by tetramethyl rhodamine ethyl ester (TMRE) staining, as per the manufacturer’s instructions (Abcam, ab113852). MitoTracker Green FM was used to measure mitochondrial abundance according to the manufacturer’s instructions (Thermo Fisher Scientific, M7514).

### ATP assay using Cell-Titer-Glo

Cell-Titer-Glo (#G7570) was obtained from Promega, Inc., and was used according to the manufacturer’s recommendations to measure ATP levels in cells. Luminescence content was evaluated using the using the Promega GloMax plate reader. All data were normalized to cell number with Quant-iT PicoGreen dsDNA Assay Kit (Invitrogen).

### Animal studies

All experiments involving mice were approved by the CWRU Institutional Animal Care Regulations and Use Committee (IACUC, protocol 2018-0063). Ten-week-old female athymic nude mice (Nude-Foxn1nu) were purchased from Harlan Laboratories (6903 M). A375 cells, were suspended in 150 µL solution comprised of 50% Dulbecco’s PBS and 50% Matrigel. Suspensions of 1 × 10^6^ cells were injected subcutaneously into the right flank of mice. For syngeneic orthotopic experiments, 1 × 10^5^ YUMM1.7 and 4 × 10^4^ B16-F10 cells were suspended in the same manner and injected into flanks of immunocompetent eight week-old C57BL/6 J mice.

Treatments were initiated after tumors were first palpable and reached 100–120 mm^3^ (nude mice) or 80–100 mm^3^ (C57BL/6 J mice). Phenformin (MedChemExpress, HY-16397 A) was dissolved in sterile water and administered orally at 50 mg/kg, five times per week. IACS-010759 (MedChemExpress, HY-112037) was formulated in 0.5% methylcellulose and delivered orally at 5 mg/kg, five times per week. Temozolomide (TMZ; Sigma-Aldrich, T2577) was freshly dissolved in DMSO and diluted in sterile saline for intraperitoneal injection, administered at 30 mg/kg, five times per week. Cisplatin (Sigma-Aldrich, 479306) was dissolved in sterile 0.9% saline and given intraperitoneally at 5 mg/kg once per week.

Body weights were measured weekly and tumor volumes were measured twice a week. For the latter, Vernier calipers were utilized and volumes were estimated by the formula, Volume = (Length × Width^2^)/2. At the end of the experiment, mice were euthanized by carbon dioxide inhalation and tumors were immediately resected for additional studies and stored at − 80 °C.

### Statistical analysis

Data were expressed as mean ± SEM (standard error of the mean) of at least three independent experiments unless indicated. Comparisons between groups were determined using an unpaired, two-tailed Student *t-*test (* *p* < 0.05; ** *p* < 0.01; *** *p* < 0.001 **** *p* < 0.0001). Statistical analyses were performed with GraphPad Prism 10.0.2 software.

## Results

### Mitochondrial respiratory profile of melanoma cell lines

**Fig. 1:**
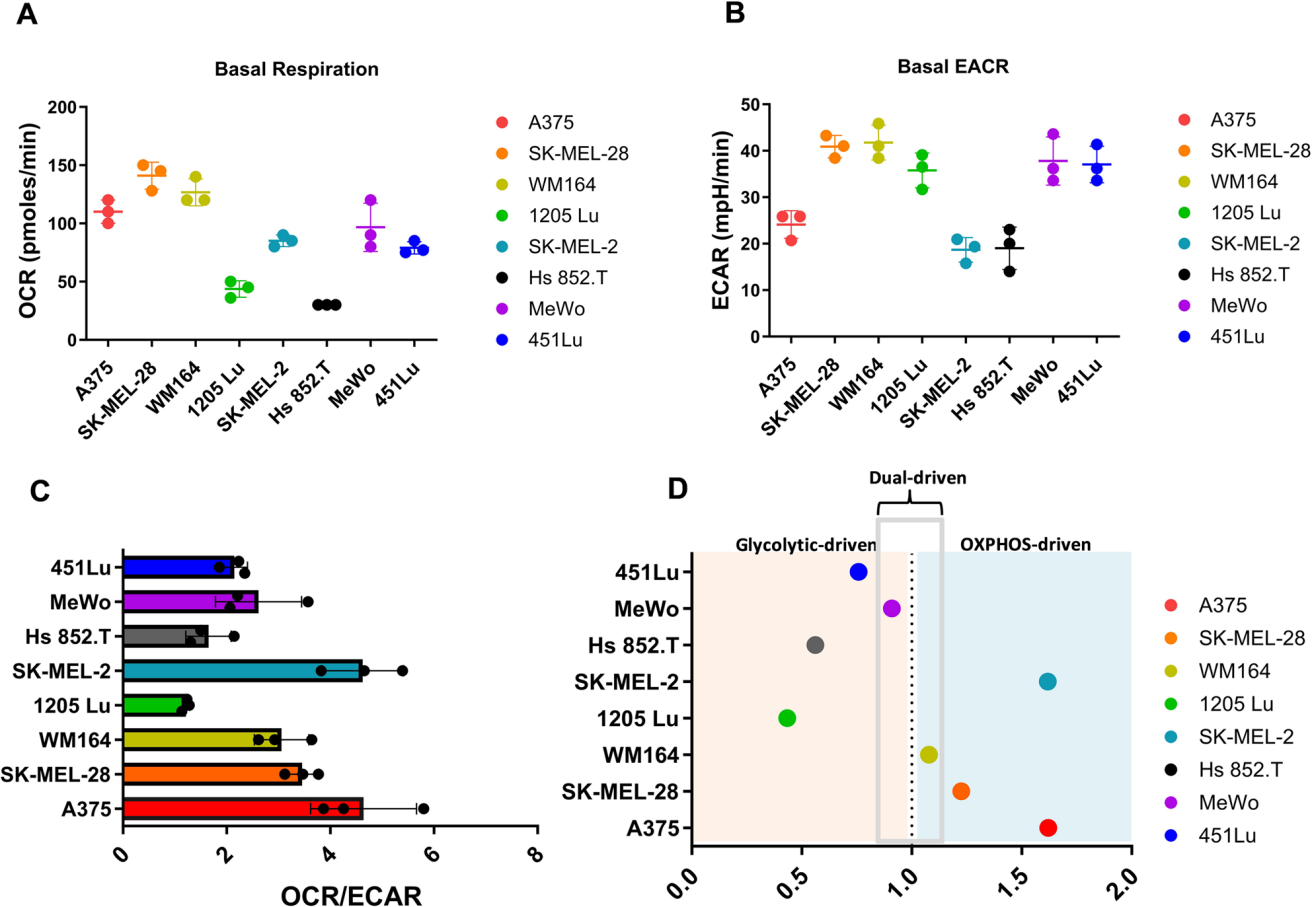
Energetic metabolism in melanoma cell lines. **A** & **B** Basal mitochondrial oxygen consumption rate (OCR) and extracellular acidification rate (ECAR) in eight different melanoma cell lines. Cells were treated with mitochondrial inhibitors as indicated: oligomycin (Oligo), carbonyl cyanide-p-trifluoromethoxyphenyl-hydrazon (FCCP), antimycin A and rotenone (Anti/Rot). **C** OCR/ECAR ratio in melanoma cell lines, representing metabolic dependencies at baseline. **D** Classification of cell lines as OXPHOS-driven or glycolytic-driven based on median OCR and ECAR values. Representative profiles of at least three replicates are shown

Oxygen consumption rate (OCR) and extracellular acidification rate (ECAR) was measured in eight different melanoma cell lines and a range of metabolic phenotypes was observed (Fig. 1A, B). To better characterize relative mitochondrial robustness, we computed OCR/ECAR ratios (Fig. 1C). Median values across all cell lines were used as thresholds to define “high” and “low” mitochondrial activity. Based on these ratios, cell lines were ranked as OXPHOS-driven or glycolytic-driven (Fig. 1D), highlighting the metabolic heterogeneity of melanoma in cell line models.

### Enhanced TCA activity and respiration in response to chemotherapy treatment

**Fig. 2:**
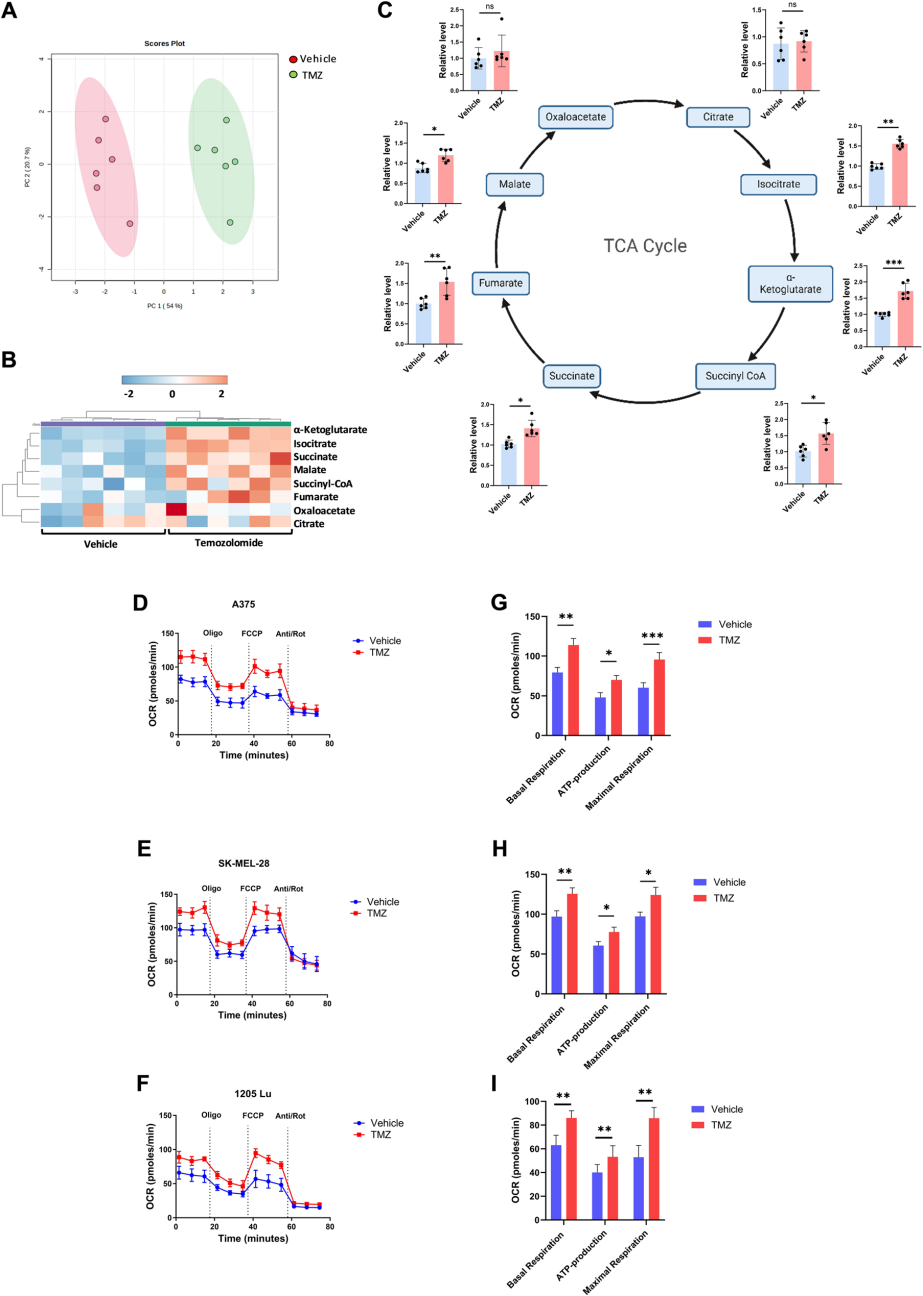
Chemotherapy administration increases TCA cycle related metabolites and cellular bioenergetics. **A** Principal component (PC) analysis was conducted in order to compare the overall metabolomics profiles in harvested cells after treatment with control and TMZ (*n* = 6 samples) in the A375 cell line. **B** A heatmap of TCA cycle metabolites after treated with control versus TMZ (*n* = 6 samples). The scale is log 2 fold-change. **C** Representative diagram of TCA cycle showing the relative metabolite levels following treatment with TMZ as measured by LC-MS/MS (*n* = 6 samples). Elevation in TCA cycle metabolites are seen in the TMZ treated group after 48 h. **D** A375, **E** SK-MEL28 and **F** 1205Lu - Representative oxygen consumption rate (OCR) in melanoma cell lines cultured with and without TMZ for 36 h prior to Seahorse analysis. Treatment with mitochondrial inhibitors is indicated: oligomycin A (Oligo), cyanide-p- trifluoromethoxyphenyl-hydrazone (FCCP), antimycin A and rotenone (Anti/Rot). **G** A375 H] SK-MEL-28 and **I** 1205Lu - Quantitative analysis of basal mitochondrial respiration, ATP production, and maximal mitochondrial respiration from the kinetic profiles of OCR. Each data point represents the mean ± SEM of at least three independent experiments. N.S., nonsignificant; *, *P* < 0.05; **, *P* < 0.01; ***, *P* < 0.001

We treated melanoma cells with a sublethal dose (IC_30_) of TMZ to identify the biological effects on cellular metabolism, independent of apoptosis or other mechanisms of cell death. Liquid chromatography coupled tandem-mass-spectrometry (LC-MS/MS) metabolomics revealed significant metabolic reprogramming following TMZ treatment. Hierarchical clustering analysis (HCA) and heatmap showed a large metabolic shift with TMZ treatment, with the top 50 most altered metabolites demonstrating widespread increases in metabolite levels in treated cells compared to controls (Supplemental Fig. S1A and S1B). Focusing on mitochondrial metabolism, principal component analysis (Fig. 2A) confirmed clear separation between the treated and untreated groups. HCA and heatmap profiling of TCA cycle metabolites (Fig. 2B) demonstrated a clear increase in mitochondrial TCA cycle intermediates after TMZ treatment. Pooled abundances of specific metabolites show roughly 1.5-fold increases across all TCA metabolites, with the exception of citric acid and oxaloacetate, likely due to their rapid turnover (Fig. 2C). Pathway enrichment analysis identified multiple specific pathways, impacted by TMZ treatment (beyond TCA) including amino acid metabolism, antioxidant and pyrimidine metabolism (Supplemental Fig. S1C). Moreover, Seahorse extracellular flux analysis demonstrated enhanced mitochondrial function in TMZ-treated cells. At IC_50_ doses (Supplemental Fig. S2A and S2B), TMZ significantly increased basal oxygen consumption rate (OCR), ATP production, and maximal respiratory capacity in both OXPHOS-driven and glycolytic-driven cell lines, indicating that chemotherapy enhances OXPHOS even in glycolytic melanoma phenotypes, as compared to untreated control cells (Fig. 2D-I). These findings suggest that TMZ induces a metabolic shift toward enhanced mitochondrial respiration and TCA cycle activity, potentially contributing to adaptive responses across multiple melanoma cell lines.

### Enrichment of mitochondrial respiratory complex I in response to chemotherapy in melanoma

**Fig. 3:**
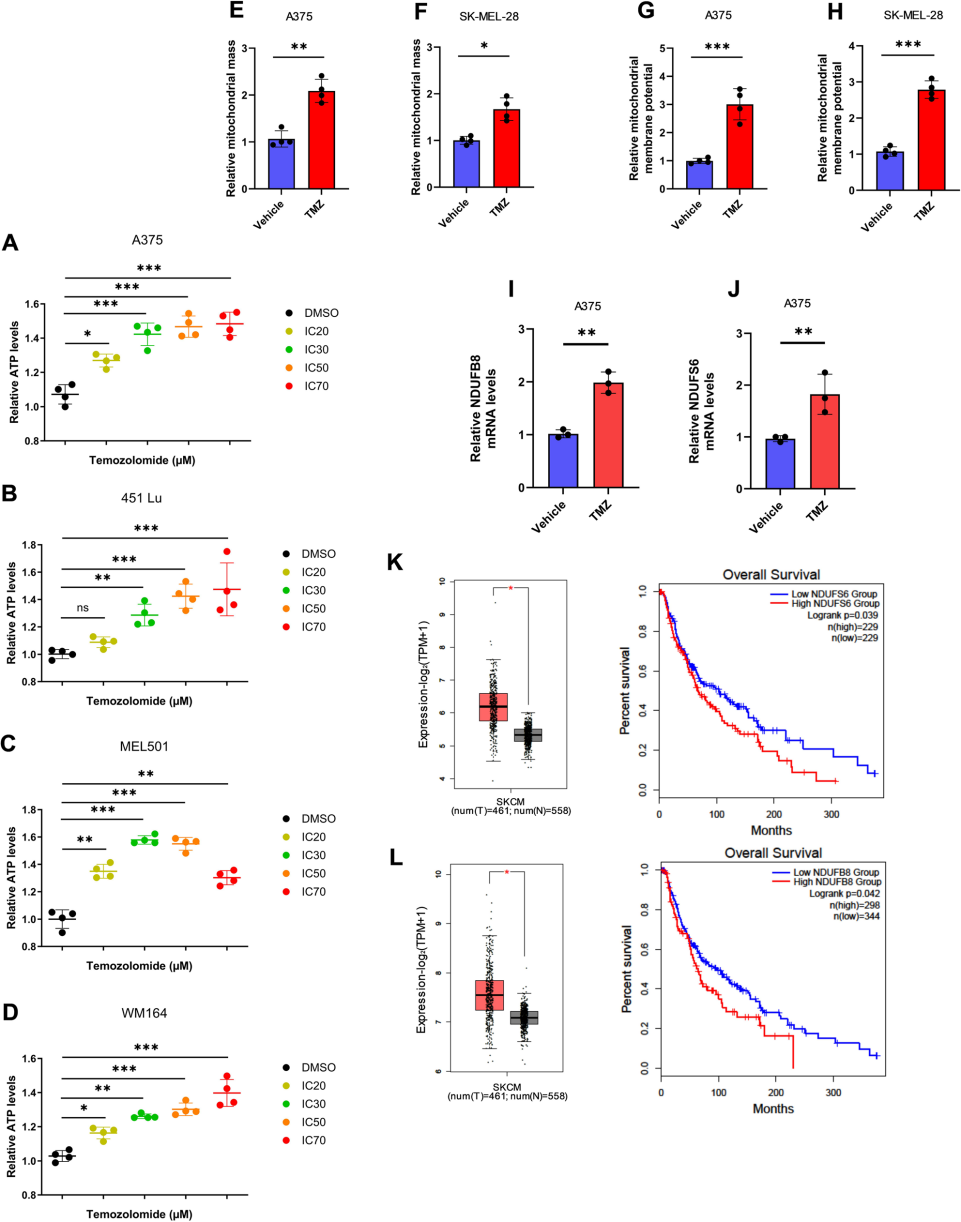
Temozolomide enhances mitochondrial content and OXPHOS. **A**-**D** ATP production level in melanoma cells treated with increasing inhibitory concentrations of TMZ. **E** A375 and **F** SK-MEL-28 - Mitochondrial mass (MitoTracker Green FM) of melanoma cells were measure 36 h after treating with the cells with TMZ. **G** A375 and **H** SK-MEL-28 - The mitochondrial membrane potential (ΔΨm), tetramethylrhodamine, ethyl ester (TMRE) of melanoma cells were measured 36 h after treating the cells with TMZ. **I**-**J** qPCR analysis of NDUFS6 and NDUFB8 in A375 and SK-MEL-28 cells after 48 h of treatment with TMZ. Expression levels are normalized to 18 S expression in each cell line. **K**–**L** RNA sequencing data obtained from TCGA showing expression of Complex I components, NDUFS6 and NDUFB8 melanoma samples compared to normal skin tissue. Association between NDUFS6 and NDUFB8 expression and overall survival, analyzed using the Kaplan–Meier. Each data point represents the mean ± SEM of at least three independent experiments. N.S., nonsignificant; *, *P* < 0.05; **, *P* < 0.01; ***, *P* < 0.001

Since melanoma cells adapt to cancer-associated stressors, such as nutrient depletion, through enhanced mitochondrial structure and function [23], we examined if chemotherapeutic stress resulted in augmented mitochondrial structure and function, beyond the observed changes in metabolomics and cellular respiration. In different melanoma cell lines, TMZ resulted in increased ATP production (Fig. 3A-D) at different IC doses (Supplemental Fig. S2A-F), and generally in direct proportion to rising doses. Comparable results were observed with cisplatin (Supplemental Fig. S3A-S3D). Additionally, two separate cell line analyses revealed augmented mitochondrial abundance, by MitoTracker Green FM (Fig. 3E and F). Similarly, tetramethylrhodamine, ethyl ester (TMRE) staining showed an increase in overall mitochondrial membrane potential (ΔΨm), demonstrating improved polarization capabilities after TMZ treatment (Fig. 3G and H). The mitochondrial import receptor sublocalized to the outer mitochondrial membrane, TOM20, was also elevated with chemotherapy treatment (Supplemental Fig. S2E and S2F). Consistent with this notion, examination of mRNA from both melanoma cell lines treated with TMZ demonstrated a high expression level of two complex I-associated transcripts, NDUFS6 and NDUFB8, compared to control (Fig. 3I and J). These findings were also observed in cell lines treated with cisplatin (Supplemental Fig. S3E and S3F). The TCGA database demonstrated strong evidence that NDUFS6 and NDUFB8 are highly expressed in melanoma samples, as compared to normal skin tissue, perhaps a function of baseline cancer-associated stress preferentially inducing mitochondrial-associated proteins. Further, higher tumoral expression of NDUFS6 and NDUFB8 were associated with worse overall survival, among patients with melanoma (Fig. 3K and L).

### Targeting mitochondrial ETC complex I sensitizes melanoma cells to chemotherapy

**Fig. 4:**
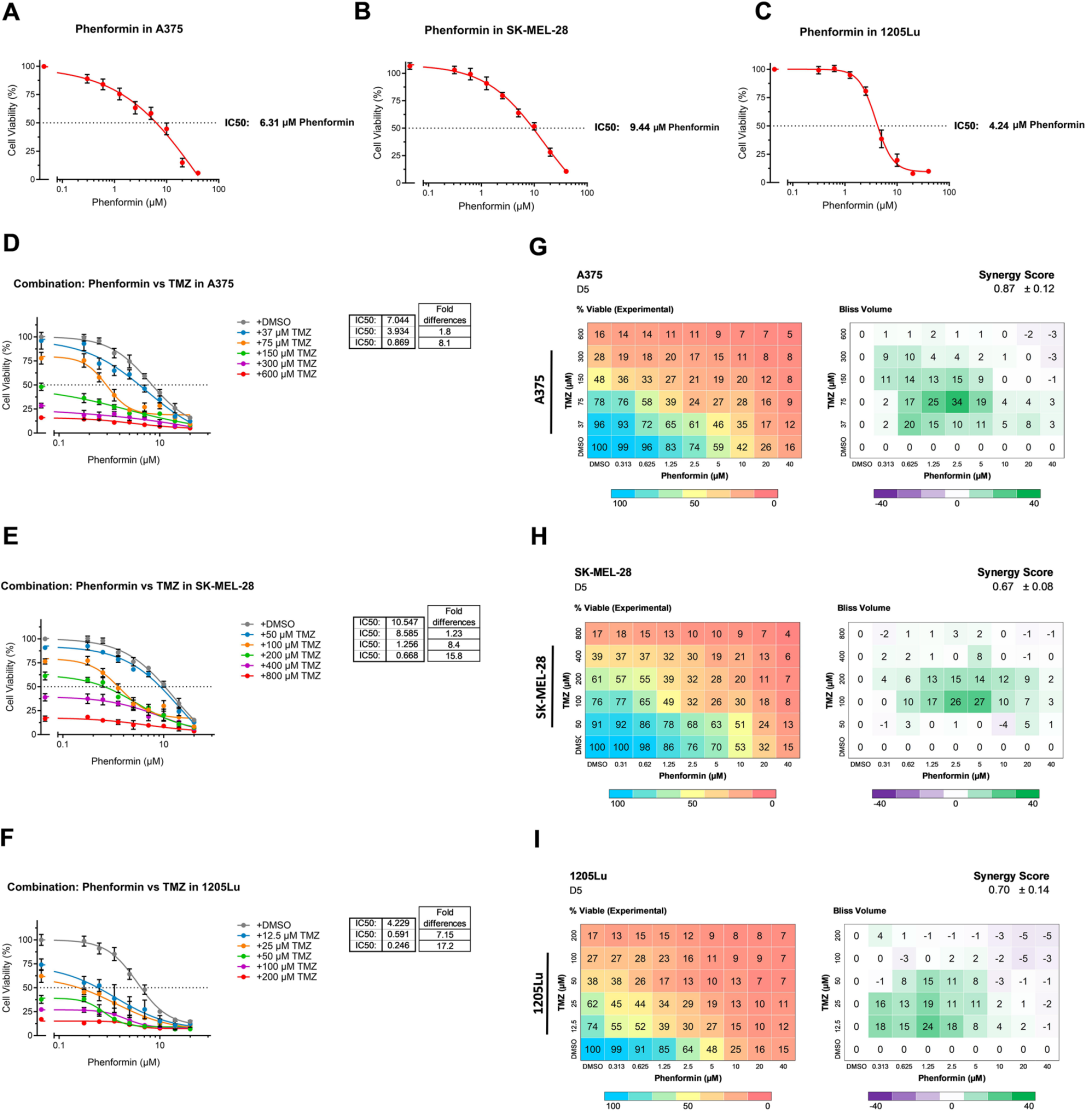
Targeting Complex I by phenformin sensitizes melanoma cells to chemotherapy. **A**-**C** Cell viability of the A375, SK-MEL-28 and 1205Lu cell lines treated with the indicated doses of phenformin. IC_50_ values are provided. **D**, **E** and **F** Drug sensitivity in A375, SK-MEL-28 and 1205Lu with varying concentrations of phenformin and temozolomide, cultured for 5days. **G**, **H** and **I** Drug matrix heatmap 6 × 8 (temozolomide and phenformin) grid showing percent viability and Bliss Independence scores in A375, SK-MEL-28 and 1205Lu cells cultured for 5 days. Positive values reflect synergy and appear green on the right-sided heatmap

Given the observed metabolic reprogramming and the enrichment of mitochondrial respiratory Complex I following chemotherapy, we hypothesized that targeting mitochondrial Complex I, a metabolic dependency, would synergize with TMZ and offer a strategy to break chemotherapy-associated metabolic resistance [42, 43]. Single-agent dose-response studies using relevant drugs were first performed to determine appropriate dosing ranges for combination studies (Fig. 4A-C). The addition of phenformin substantially increased TMZ potency (up to 8-fold, 15-fold and 17-fold in the cell lines) by PicoGreen DNA quantitation (Fig. 4D-F). Pharmacologic inhibition of complex I using phenformin revealed strong synergy with TMZ by Bliss index analyses, as reflected in the positive Synergy Scores for both cell (0.87, 0.67 and 0.70; >0 reflects synergy) (Fig. 4G-I). Notably, the most synergy occurs around the IC30-50 doses of TMZ and phenformin.

### Targeting mitochondrial ETC complex I enhances melanoma sensitivity to chemotherapy in vivo

**Fig. 5:**
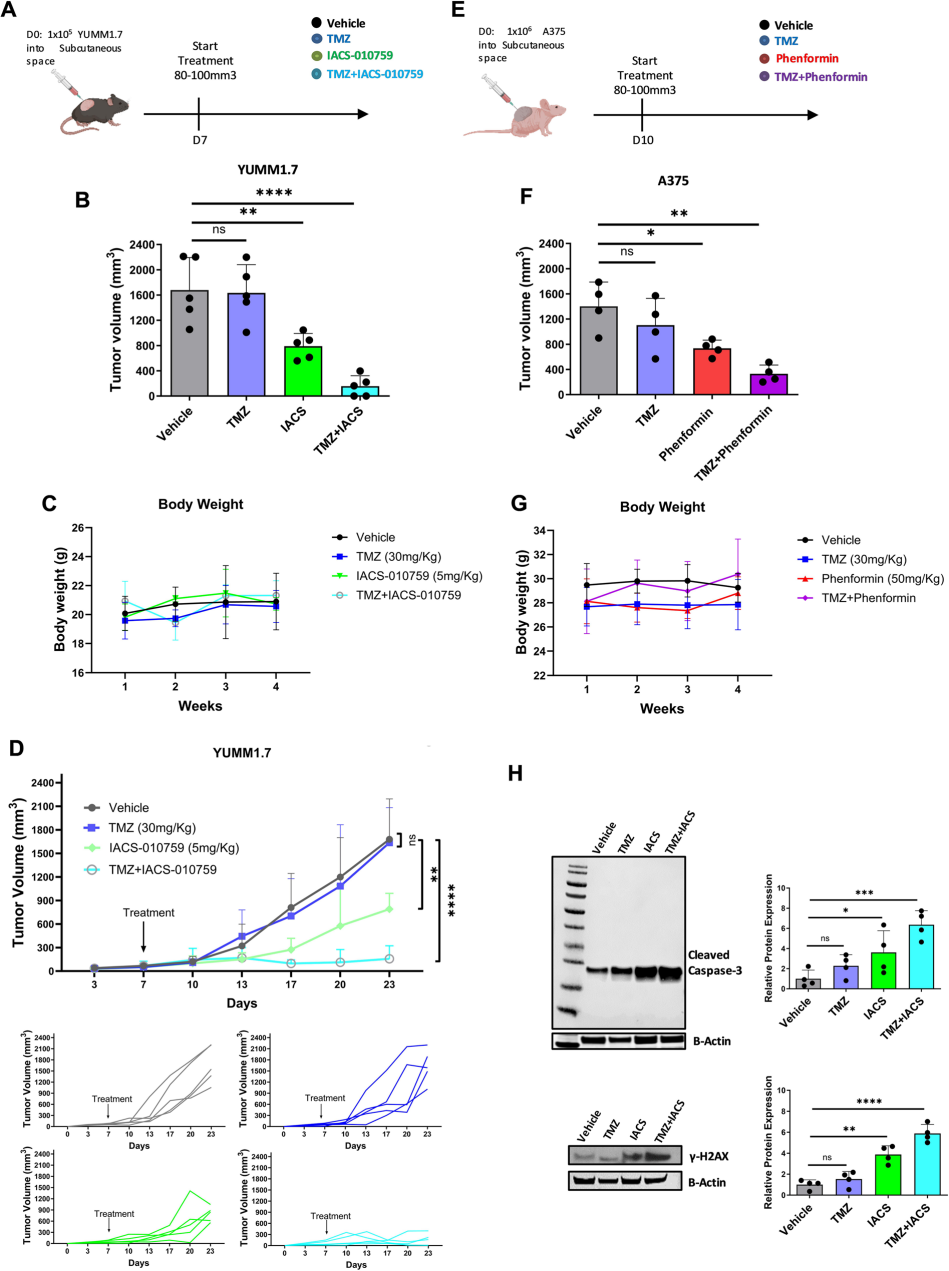
Targeting mitochondrial ETC complex I enhances melanoma cells sensitivity to chemotherapyin vivo.**A** Schematic of the treatment model in which 1 × 10^5^ YUMM1.7 melanoma cells were injected subcutaneously into the flank C57BL/6 mice. After 8–10 days, when tumors reached 80–100 mm3, mice were divided into four groups and treated with (i) Vehicle; (ii) TMZ (30 mg/kg) every day; (iii) IACS-010759 (5 mg/kg) once a day; (iv) IACS-010759 + TMZ (5 mg/kg + 30 mg/kg). **B** Average tumor volume at the end of the experiment (*n* = 5 tumors per group). **C** Body weights of C57BL/6 mice bearing YUMM1.7 tumors throughout the study (*n* = 5 per group). **D** Tumor growth curves of individual YUMM1.7 tumors in C57BL/6 mice. Tumor volumes were measured twice weekly using calipers (*n* = 5 per group). **E** Schematic representation of the treatment model after 1 × 10^6^ A375 melanoma cells were injected subcutaneously into the flank of nude mice. After 8–10 days, when tumors reached 80–100 mm3, mice were divided into four groups and treated with (i) Vehicle; (ii) TMZ (30 mg/kg) every day; (iii) Phenformin (50 mg/kg) once a day; (iv) phenformin + TMZ (50 mg/kg + 30 mg/kg). **F** Average tumor volume at the end of the experiment (*n* = 4 tumors per group). **G** Body weights of nude mice bearing A375 xenografts (*n* = 4 per group). **H** Representative immunoblot analysis of cleaved caspase-3 and γH2AX expression in tumor samples, with beta-actin used for normalization of cellular protein. The relative protein levels were quantified by densitometry. The full set of tumor immunoblots is provided in the Supplementary Figure. The full set of tumor immunoblots is provided in Supplementary Figure S4H. Each data point represents the mean ± SEM of at least three independent experiments. N.S., nonsignificant; *, *P* < 0.05; **, *P* < 0.01; ****, *P* < 0.0001

To evaluate the therapeutic efficacy of Complex I inhibition in an immunocompetent setting, we conducted two separate experiments using syngeneic C57BL/6J mice implanted with YUMM1.7 murine melanoma cells. In the first experiment, mice were treated with vehicle control, temozolomide (TMZ; 30 mg/kg intraperitoneally, once daily), IACS-010759 (5 mg/kg orally, once daily), or the combination of IACS-010759 + TMZ (5 mg/kg orally + 30 mg/kg intraperitoneally, once daily). IACS-010759 monotherapy showed greater efficacy than TMZ alone, and the combination treatment significantly reduced tumor growth compared to either agent alone (Fig. 5A–D). All treatments were well tolerated, with no significant body weight loss observed. In a separate experiment, we tested phenformin (50 mg/kg orally, once daily), either alone or in combination with TMZ (50 mg/kg orally + 30 mg/kg intraperitoneally, once daily), alongside vehicle and TMZ-only controls. As with IACS-010759, phenformin alone outperformed TMZ, and the combination therapy achieved the greatest tumor suppression (Supplemental Fig. S4A–D). These treatments were also well tolerated.

To determine whether this chemosensitizing effect extended to additional chemotherapy agents and tumor models, we performed a parallel study using the B16-F10 melanoma model. Mice were treated with vehicle, cisplatin (5 mg/kg), phenformin (50 mg/kg), or phenformin + cisplatin. Similarly, the combination therapy showed the strongest tumor growth inhibition (Supplemental Fig. S4E–G). Body weights remained stable across groups, indicating good tolerability.

To further validate these findings in a human melanoma model, we employed nude mice bearing A375 xenografts (Fig. 5E). Mice were treated with phenformin, TMZ, or their combination. Consistent with the findings in the immunocompetent model, combination therapy resulted in significantly enhanced anti-tumor activity compared to monotherapies (Fig. 5F). Importantly, no signs of treatment-related distress were observed, as indicated by stable body weights across all groups (Fig. 5G). Furthermore, tumors from mice receiving combination treatment exhibited increased apoptosis and DNA damage, as indicated by elevated levels of cleaved caspase-3 protein and γH2AX (Fig. 5H). TMZ alone caused only modest cleaved caspase-3 without γH2AX, suggesting mitochondrial stress rather than full apoptosis, whereas the combination triggered robust apoptotic signaling. Taken together, these findings highlight Complex I as a key metabolic vulnerability in melanoma, especially in combination with chemotherapy. The addition of a Complex I inhibitor appears to reduce chemotherapy resistance.

## Discussion

The role of mitochondrial OXPHOS has garnered increasing attention as a key metabolic pathway in melanoma because of its impact on therapeutic efficacy [44]. While immune checkpoint inhibitors and targeted therapies have revolutionized the treatment landscape for metastatic melanoma, a considerable subset of patients either fail to respond to these treatments or experience significant toxicity. For these patients, cytotoxic chemotherapy remains a last-resort salvage option [45]. The limited efficacy of conventional chemotherapeutic approaches underscores the need for novel strategies to sensitize melanoma cells to chemotherapy, as combination therapies might expand treatment options for patients ineligible for immune or targeted therapies. Preclinical studies have indicated that targeting metabolic pathways, including mitochondrial bioenergetics, holds promise in reversing resistance to conventional therapies [46–49].

While metabolic plasticity and heterogeneity are well-recognized features of melanoma, our study provides novel insight into how these traits directly influence chemotherapy response and sensitivity to mitochondrial Complex I inhibition, offering a more translationally relevant perspective. We show that melanoma cells adapt to chemotherapy-induced stress by upregulating mitochondrial OXPHOS, a transition marked by increased TCA cycle intermediates, elevated OXPHOS activity, and enhanced mitochondrial content. These findings support the idea that increased mitochondrial metabolism serves as a critical adaptive mechanism that promotes melanoma cell survival under cytotoxic stress. Although the cell lines varied in their baseline metabolic phenotypes, these differences did not predict sensitivity to TMZ, as no consistent association was observed across either OXPHOS- or glycolytic-driven melanoma cell lines.

To counteract the metabolic adaptations driving chemotherapy resistance, we investigated the therapeutic potential of targeting OXPHOS to disrupt melanoma survival mechanisms. Inhibitors of the mitochondrial ETC complexes, such as phenformin and IACS-010759, have been shown to decrease OXPHOS activity and increase susceptibility to apoptosis [50, 51]. Our study demonstrates synergy between the chemotherapy drug TMZ and these ETC complex inhibitors, significantly enhancing tumor cell death across multiple melanoma cell lines. This effect was further observed in vivo, where both phenformin and IACS-010759 noticeably enhanced the antitumor efficacy of TMZ in melanoma tumors.

A potential mechanism underlying this synergy is that heightened mitochondrial respiration in response to TMZ sustains stress resistance, a pro-survival process that is disrupted by OXPHOS inhibition. Pharmacological suppression of adaptive mitochondrial bioenergetics induces a metabolic shift toward an energetically compromised state, rendering melanoma cells more vulnerable to chemotherapy-induced cytotoxicity. These findings highlight mitochondrial metabolism as a key therapeutic target and support the clinical potential of combining mitochondrial inhibitors with chemotherapy to overcome metabolic adaptations that drive drug resistance in melanoma.

Clinical interest in targeting mitochondrial Complex I in melanoma has led to the exploration of agents such as phenformin and IACS-010759. Phenformin, a more potent biguanide than metformin, has demonstrated preclinical efficacy in melanoma, particularly in tumors with high OXPHOS dependency or resistance to BRAF inhibitors [52]. However, its clinical application has been limited by the risk of lactic acidosis, especially in patients with renal impairment. Early-phase trials combining phenformin with targeted therapies have shown some signs of tolerability at reduced doses, though efficacy data remain limited [53]. IACS-010759, a selective Complex I inhibitor, is currently under clinical evaluation in solid tumors and hematologic malignancies, with early data indicating manageable toxicity and biological activity [51]. While clinical experience in melanoma remains early, these findings support the rationale for further investigation into OXPHOS-targeting strategies, particularly in combination with chemotherapy, as a means to overcome resistance and expand treatment options for patients with limited therapeutic alternatives.

## Conclusions

This study reinforces the pivotal role of metabolic reprogramming in melanoma chemotherapy resistance. Our findings highlight the crucial role of TCA cycle metabolites in maintaining cellular homeostasis in melanoma and reveal plasticity and specific adaptations in response to anticancer chemotherapy. Inhibition of mitochondrial ETC subunits resulted in increased cell death, especially when combined with conventional anti-melanoma cytotoxic chemotherapies. Future research, should prioritize in vivo validation and clinical trials which will be essential to confirm the therapeutic potential of combining OXPHOS inhibitors with chemotherapy in melanoma. This approach holds promise for overcoming chemotherapy resistance and expanding treatment options for patients who are refractory to current standard therapies.

## Supplementary Information

Supplementary Material 1: Supplemental Figure-1. Chemotherapy-induced metabolic changes and pathway alterations. A] Heatmap showing the relative abundance of ∼299 metabolites analyzed by LC/MS-MS performed on A375 cells treated with TMZ or CTRL for 36 hours (n = 6 samples). B] Top 50 metabolites altered on cells treated with TMZ or CTRL. C] Metabolic pathway analyses of altered metabolites; bars are colored according to P values, and the bar length is based on fold enrichment. Supplemental Figure-2 Inhibitory concentration determination for temozolomide on melanoma cells and mitochondrial membrane potential. A-F] Cell viability of the A375, SK-MEL-28, SK-MEL-2, and 1205Lu, WM164 and 451Lu treated with TMZ. IC20, IC30, IC50 and IC70 values are provided. G-H] Representative immunoblot analysis of TOM20 expression in A375 and SK-MEL-28 cells after 72 hours of treatment with IC30 concentration of TMZ, beta-actin used for normalization of cellular protein. The relative protein expression level of TOM20 across three independent experiments is quantified by densitometry. Each data point represents the mean ± SEM of at least three independent experiments. N.S., nonsignificant; *, P < 0.05; **, P < 0.01; ***, P < 0.001; ****, P < 0.0001.. Supplemental Figure -3 cisplatin enhances mitochondrial content and OXPHOS. A-B] Cell viability of the A375 and WM164 cell lines treated with the indicated doses of cisplatin. IC20, IC30, IC50 and IC70 values are provided. C-D] ATP production level in melanoma cells treated with increasing inhibitory concentrations of TMZ. E-F] qPCR analysis of NDUFS6 and NDUFB8 in A375 cells after 48 hours of treatment with cisplatin. Supplemental Figure -4 Targeting mitochondrial ETC with phenformin enhances melanoma cells sensitivity to chemotherapy in vivo in C57BL/6 mice. A] Schematic of the treatment model in which 1 × 105 YUMM1.7 melanoma cells were injected subcutaneously into the flank C57BL/6 mice. After 8-10 days, when tumors reached 80-100 mm3, mice were divided into four groups and treated with i) Vehicle; ii) TMZ (30 mg/kg) every day; iii) phenformin (50 mg/kg) once a day; iv) phenformin+ TMZ (50 mg/kg + 30 mg/kg). B] Average tumor volume at the end of the experiment (n = 5 tumors per group). C] Body weights of C57BL/6 mice bearing YUMM1.7 tumors throughout the study (n = 5 per group). D] Tumor growth curves of individual YUMM1.7 tumors in C57BL/6 mice. E] Schematic of the treatment model in which 4 × 104 B16-F10 melanoma cells were injected subcutaneously into the flank C57BL/6 mice. After 6-8 days, when tumors reached 80-100 mm3, mice were divided into four groups and treated with i) Vehicle; ii) Cisplatin (5mg/kg) every day; iii) phenformin (50 mg/kg) once a day; iv) phenformin+ Cisplatin (50 mg/kg + 30 mg/kg). F] Body weights of C57BL/6 mice bearing B16-F10 tumors throughout the study (n = 5 per group). G] Tumor growth curves of individual B16-F10 tumors in C57BL/6 mice. Tumor volumes were measured twice weekly using calipers (n = 5 per group). Tumor volumes were calculated twice per week using calipers (n = 5 per group). H] Representative immunoblot analysis of cleaved caspase-3 and γH2AX expression across all harvested tumors. β-actin was used as a loading control. Each data point represents the mean ± SEM of at least three independent experiments. *, P < 0.05; **, P < 0.01; ***, P < 0.001; ****, P < 0.0001. Note: The control and TMZ group treatment is same as figure 5A-D.

## Data Availability

No datasets were generated or analysed during the current study.

## Abbreviations

OXPHOS: Oxidative phosphorylation
TCA: Tricarboxylic acid
ETC: Electron transport chain
TMZ: Temozolomide
OCR: Oxygen consumption rate
ECAR: Extracellular acidification rate
ΔΨm: Mitochondrial membrane potential
