# Supplementary material for: Melanoma resists chemotherapy through an adaptive mitochondrial response
Source: J Exp Clin Cancer Res. 2026 Mar 26;45:109. doi: 10.1186/s13046-026-03685-8 (PMC13141405; doi:10.1186/s13046-026-03685-8)

**Supplemental Figure-1 Chemotherapy-induced metabolic changes and pathway alterations**. **A]** Heatmap showing the relative abundance of ∼299 metabolites analyzed by LC/MS-MS performed on A375 cells treated with TMZ or CTRL for 36 hours (n = 6 samples). **B]** Top 50 metabolites altered on cells treated with TMZ or CTRL. **C]** Metabolic pathway analyses of altered metabolites; bars are colored according to P values, and the bar length is based on fold enrichment.

**Supplemental Figure-2 Inhibitory concentration determination for temozolomide on melanoma cells and mitochondrial membrane potential**

**A**-**F]** Cell viability of the A375, SK-MEL-28, SK-MEL-2, and 1205Lu, WM164 and 451Lu treated with TMZ. IC_20_, IC_30_, IC_50_ and IC_70_ values are provided. **G-H]** Representative immunoblot analysis of TOM20 expression in A375 and SK-MEL-28 cells after 72 hours of treatment with IC_30_ concentration of TMZ, beta-actin used for normalization of cellular protein. The relative protein expression level of TOM20 across three independent experiments is quantified by densitometry. Each data point represents the mean ± SEM of at least three independent experiments. N.S., nonsignificant; *, P < 0.05; **, P < 0.01; ***, P < 0.001; ****, P < 0.0001.

**Supplemental Figure -3** **cisplatin enhances mitochondrial content and OXPHOS**

**A-B]** Cell viability of the A375 and WM164 cell lines treated with the indicated doses of cisplatin. IC_20_, IC_30_, IC_50_ and IC_70_ values are provided. **C**-**D]** ATP production level in melanoma cells treated with increasing inhibitory concentrations of TMZ. **E**-**F]** qPCR analysis of NDUFS6 and NDUFB8 in A375 cells after 48 hours of treatment with cisplatin.

**Supplemental Figure -4 Targeting mitochondrial ETC with phenformin enhances melanoma cells sensitivity to chemotherapy *in vivo* in C57BL/6 mice**

**A]** Schematic of the treatment model in which 1 × 10^5^ YUMM1.7 melanoma cells were injected subcutaneously into the flank C57BL/6 mice. After 8-10 days, when tumors reached 80-100 mm3, mice were divided into four groups and treated with i) Vehicle; ii) TMZ (30 mg/kg) every day; iii) phenformin (50 mg/kg) once a day; iv) phenformin+ TMZ (50 mg/kg + 30 mg/kg). **B]** Average tumor volume at the end of the experiment (n = 5 tumors per group). **C]** Body weights of C57BL/6 mice bearing YUMM1.7 tumors throughout the study (n = 5 per group). **D]** Tumor growth curves of individual YUMM1.7 tumors in C57BL/6 mice. **E]** Schematic of the treatment model in which 4 × 10^4^ B16-F10 melanoma cells were injected subcutaneously into the flank C57BL/6 mice. After 6-8 days, when tumors reached 80-100 mm3, mice were divided into four groups and treated with i) Vehicle; ii) Cisplatin (5mg/kg) every day; iii) phenformin (50 mg/kg) once a day; iv) phenformin+ Cisplatin (50 mg/kg + 30 mg/kg). **F]** Body weights of C57BL/6 mice bearing B16-F10 tumors throughout the study (n = 5 per group). **G]** Tumor growth curves of individual B16-F10 tumors in C57BL/6 mice. Tumor volumes were measured twice weekly using calipers (n = 5 per group). Tumor volumes were calculated twice per week using calipers (n = 5 per group). **H]** Representative immunoblot analysis of cleaved caspase-3 and γH2AX expression across all harvested tumors. β-actin was used as a loading control. Each data point represents the mean ± SEM of at least three independent experiments. *, P < 0.05; **, P < 0.01; ***, P < 0.001; ****, P < 0.0001.

Note: The control and TMZ group treatment is same as figure 5A-D.


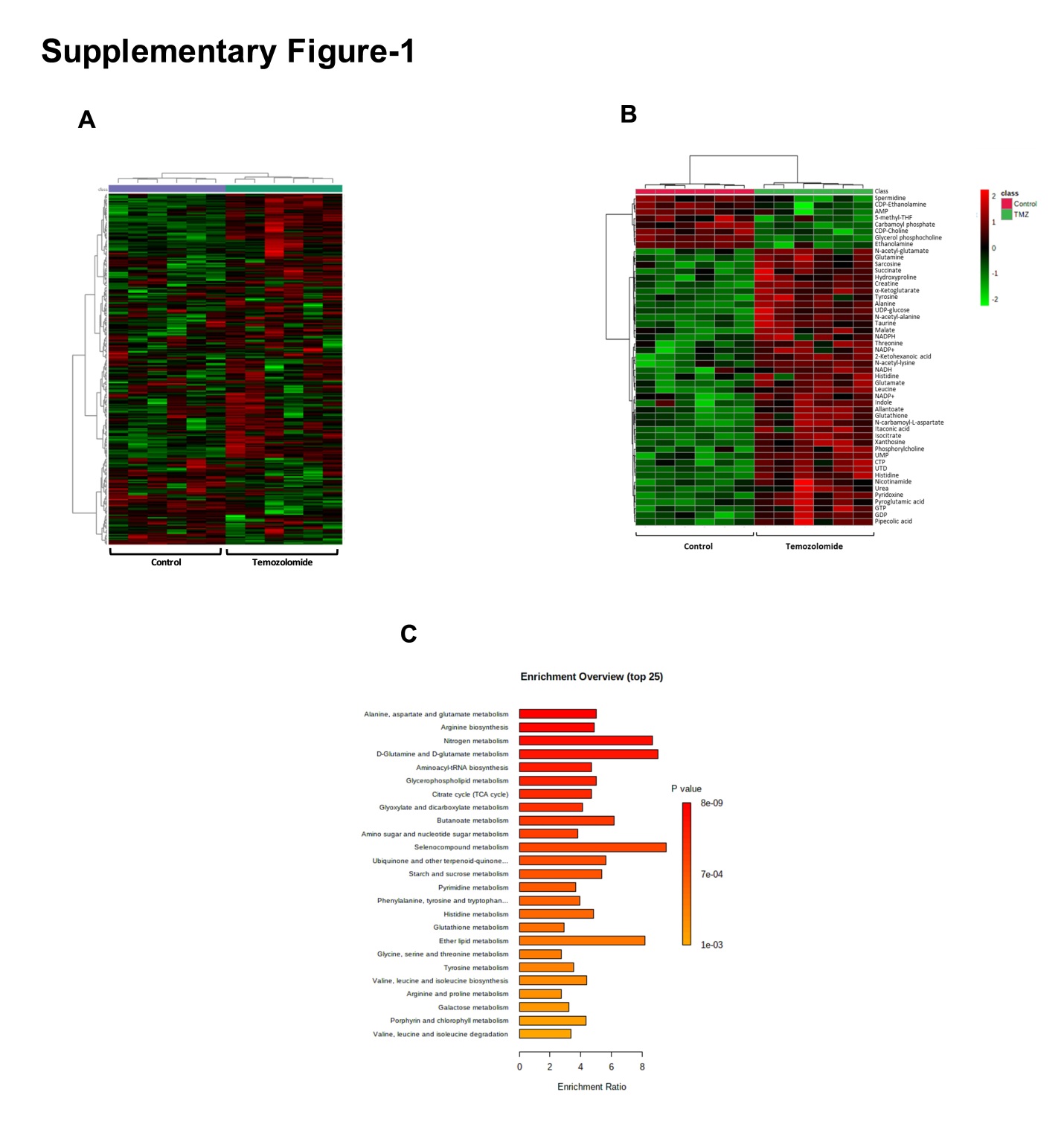


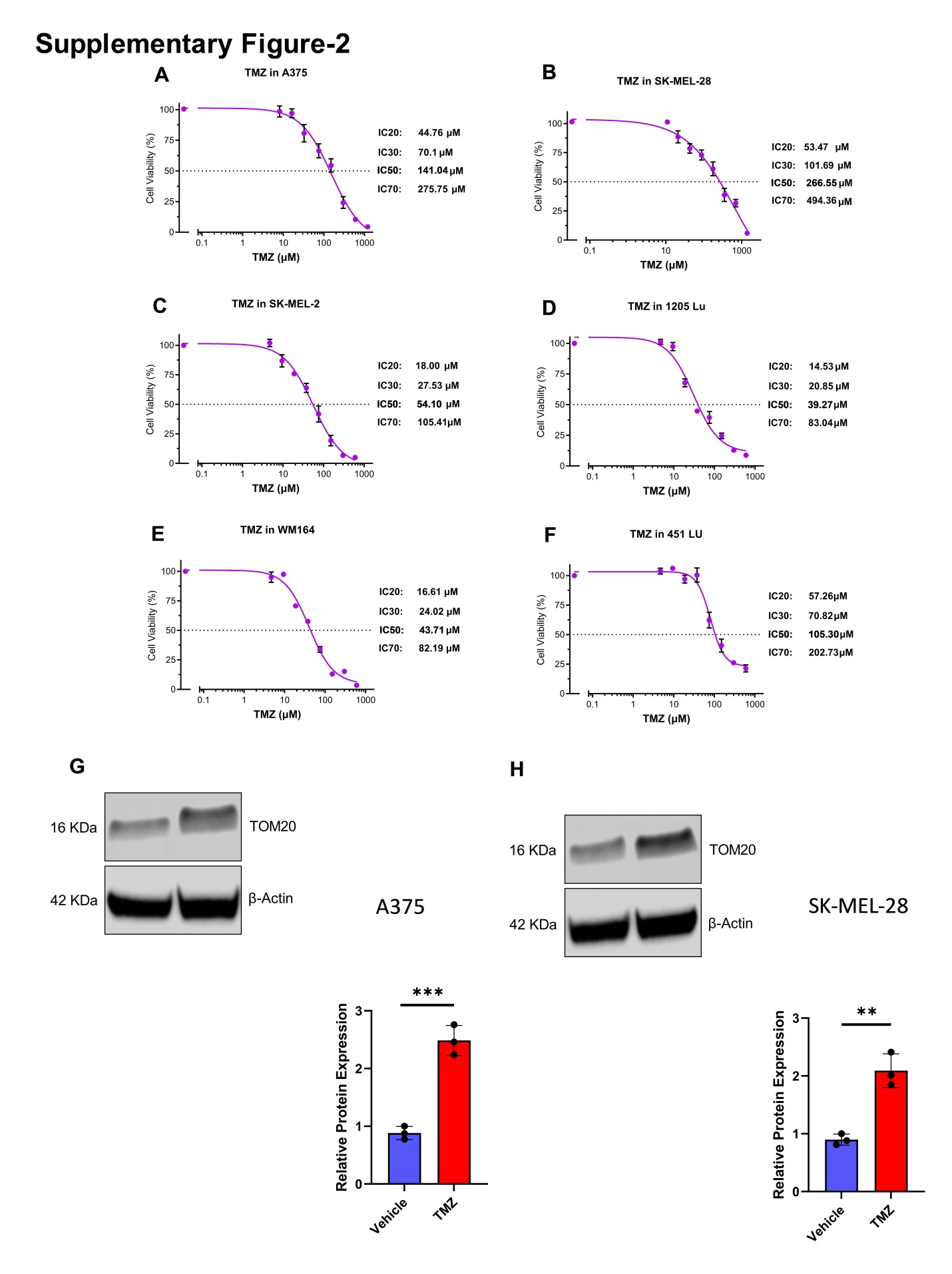


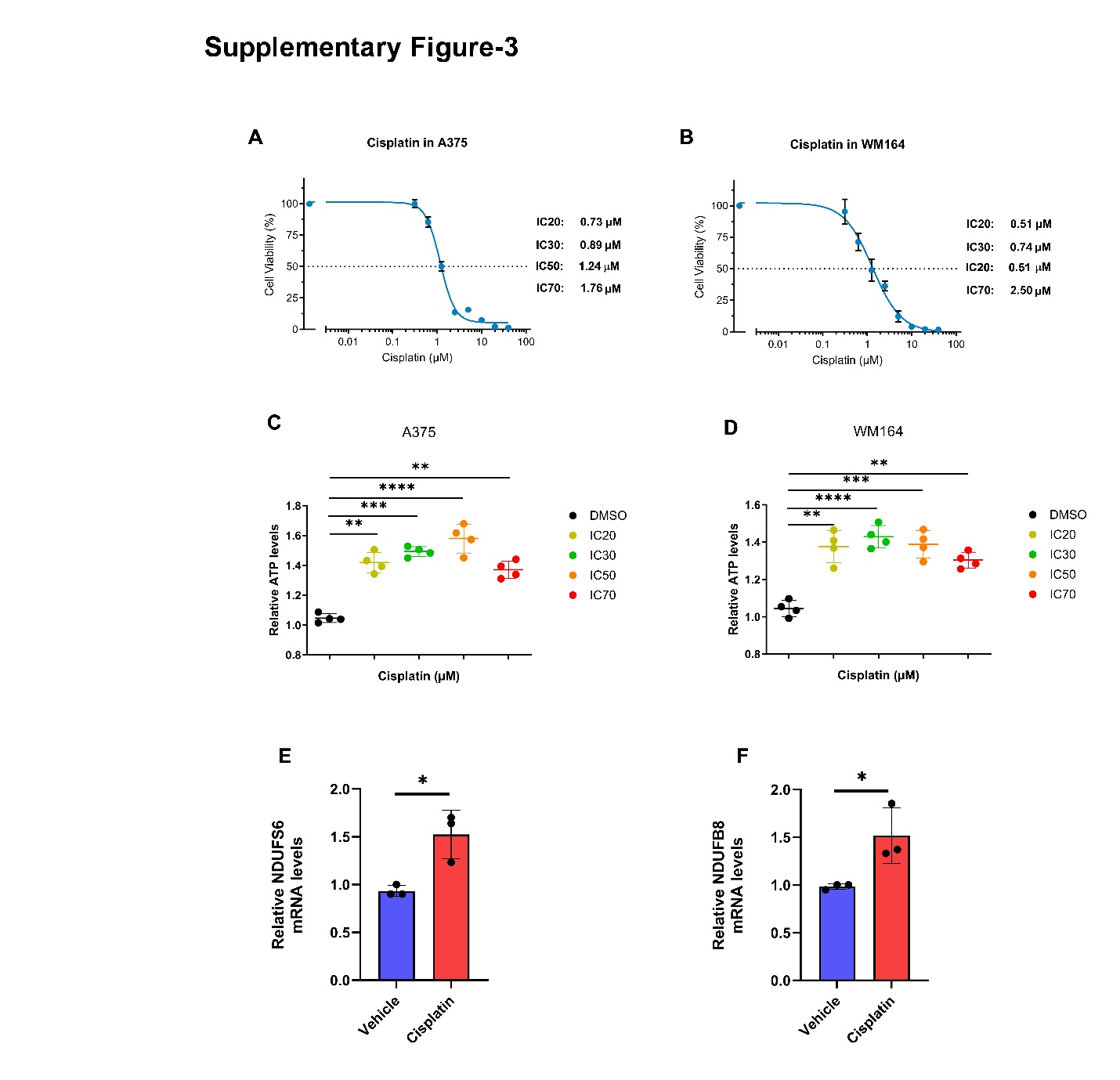


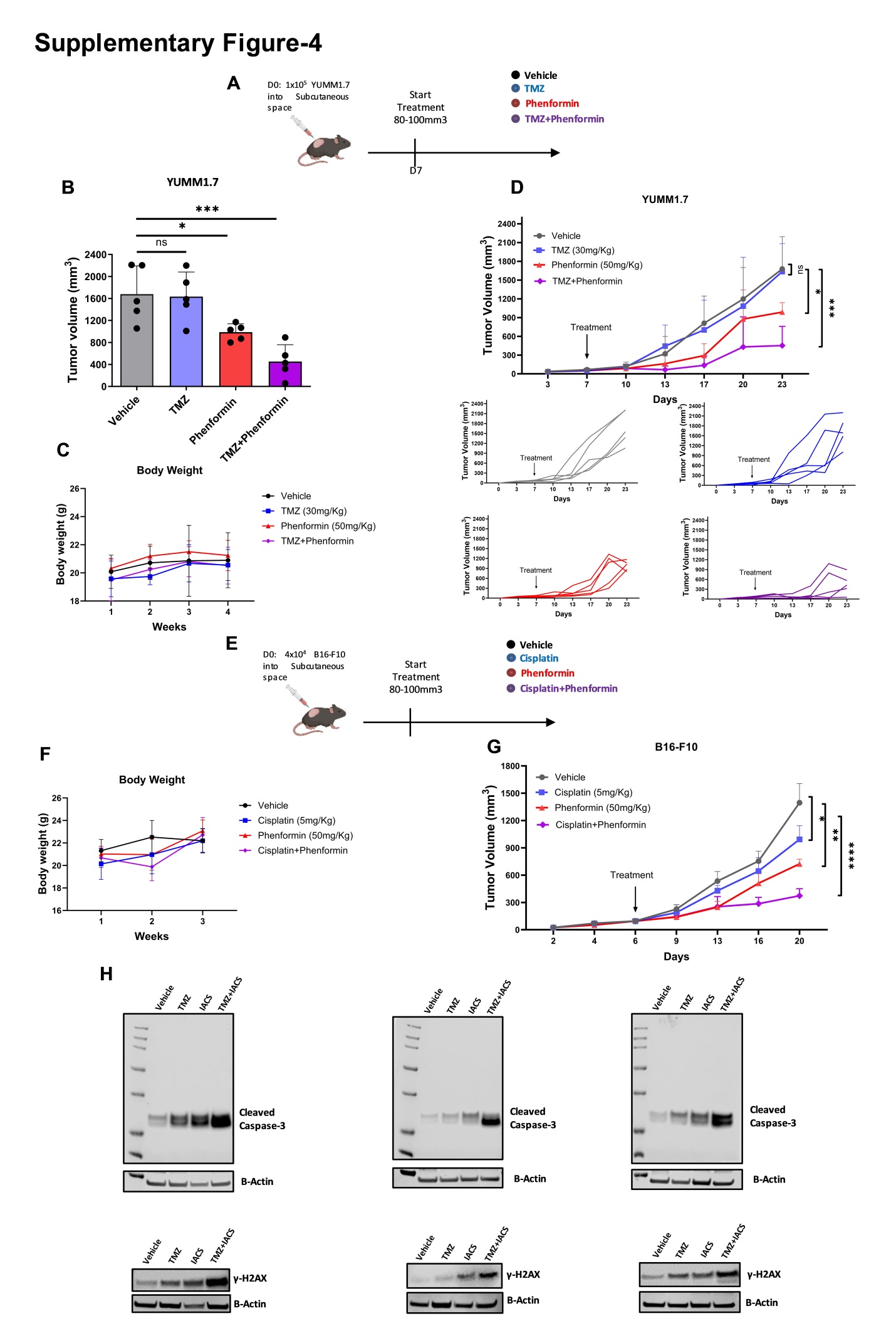

Supplement: Supplementary file 1 — Supplementary Material 1: Supplemental Figure-1. Chemotherapy-induced metabolic changes and pathway alterations. A] Heatmap showing the relative abundance of ∼299 metabolites analyzed by LC/MS-MS performed on A375 cells treated with TMZ or CTRL for 36 hours (n = 6 samples). B] Top 50 metabolites altered on cells treated with TMZ or CTRL. C] Metabolic pathway analyses of altered metabolites; bars are colored according to P values, and the bar length is based on fold enrichment. Supplemental Figure-2 Inhibitory concentration determination for temozolomide on melanoma cells and mitochondrial membrane potential. A-F] Cell viability of the A375, SK-MEL-28, SK-MEL-2, and 1205Lu, WM164 and 451Lu treated with TMZ. IC20, IC30, IC50 and IC70 values are provided. G-H] Representative immunoblot analysis of TOM20 expression in A375 and SK-MEL-28 cells after 72 hours of treatment with IC30 concentration of TMZ, beta-actin used for normalization of cellular protein. The relative protein expression level of TOM20 across three independent experiments is quantified by densitometry. Each data point represents the mean ± SEM of at least three independent experiments. N.S., nonsignificant; *, P < 0.05; **, P < 0.01; ***, P < 0.001; ****, P < 0.0001.. Supplemental Figure -3 cisplatin enhances mitochondrial content and OXPHOS. A-B] Cell viability of the A375 and WM164 cell lines treated with the indicated doses of cisplatin. IC20, IC30, IC50 and IC70 values are provided. C-D] ATP production level in melanoma cells treated with increasing inhibitory concentrations of TMZ. E-F] qPCR analysis of NDUFS6 and NDUFB8 in A375 cells after 48 hours of treatment with cisplatin. Supplemental Figure -4 Targeting mitochondrial ETC with phenformin enhances melanoma cells sensitivity to chemotherapy in vivo in C57BL/6 mice. A] Schematic of the treatment model in which 1 × 10^5^ YUMM1.7 melanoma cells were injected subcutaneously into the flank C57BL/6 mice. After 8-10 days, when tumors reached 80-100 mm3, [file 13046_2026_3685_MOESM1_ESM.docx]
